# Effect of vitamin D status on a disintegrin-like and metalloprotease with thrombospondin type 1 motif 13 (ADAMTS13) and interleukin 6 in patients with acute myeloid leukaemia

**DOI:** 10.1186/s40001-025-02879-8

**Published:** 2025-07-16

**Authors:** Dina Ashraf Abdelhady, Mervat Mostafa Mohamed Azab, Ahmed A. Alnagar, Nora Said

**Affiliations:** 1 Clinical and Chemical Pathology Department, Faculty of Medicine, Zagazig University, Zagazig, Egypt; 2 Clinical Pathology Department, Faculty of Medicine, Zagazig University, Zagazig, Egypt; 3 Medical Oncology Department, Faculty of Medicine, Zagazig University, Zagazig, Egypt

**Keywords:** AML, Inflammation, ADAMTS13, IL-6, Vitamin D, Chemotherapy

## Abstract

**Background:**

Low levels of the enzyme ADAMTS13 and elevated inflammatory markers such as interleukin-6 (IL-6) are associated with worse outcomes in patients with acute myeloid leukaemia (AML). IL-6 produced by leukaemia cells suppresses ADAMTS13 activity and impairs hematopoietic differentiation. Vitamin D, which has known for its immunomodulatory effects, may reduce IL-6 levels. This study investigated the impact of vitamin D supplementation on IL-6 and ADAMTS13 levels in patients newly diagnosed with AML.

**Patients and method:**

A total of 38 newly diagnosed AML patients and 14 healthy individuals were included. IL-6 and ADAMTS13 levels were measured in both groups at baseline. Among AML patients, 34 were found to have vitamin D deficiency. Seventeen of these patients received oral vitamin D supplementation for 28 days in addition to their standard chemotherapy. Serum levels of IL-6, ADAMTS13 and vitamin D were measured on the 1st and 28th days of chemotherapy. Statistical analyses included Mann–Whitney U test, Wilcoxon signed-rank test, and Spearman correlation.

**Results:**

AML patients had significantly higher IL-6 (7.19 vs. 1.15 pg/mL; *p* = 0.00001) and lower ADAMTS13 (684 vs. 1205 ng/mL; *p* = 0.001) compared to controls. ADAMTS13 significantly increased by Day 28 (*p* = 0.0001), while IL-6 showed no overall change. However, among vitamin D-deficient patients receiving supplementation, IL-6 levels decreased significantly by Day 28 (*p* = 0.049). A negative correlation was found between vitamin D and IL-6 on Day 28 (*r* = –0.485; *p* = 0.035). No significant effect of vitamin D status was observed on ADAMTS13 levels.

**Conclusion:**

Vitamin D supplementation was associated with a reduction in IL-6 levels, but did not influence ADAMTS13 levels in patients with AML. These findings support the potential role of vitamin D as an adjunct anti-inflammatory agent during induction chemotherapy. Further studies with larger samples and longer follow-up are recommended to clarify its therapeutic relevance.

*Trial registration number NCT05149339* in www.clinicaltrial.gov registered on 25/11/2021 'retrospectively registered’ with URL; https://register.clinicaltrials.gov/prs/app/action/SelectProtocol?sid=S000BKX8&selectaction=Edit&uid=U0005ZGQ&ts=98&cx=avh64j.

## Introduction

ADAMTS13 (a disintegrin and metalloprotease with thrombospondin type 1 domain 13) is a member of the ADAMTS family that acts to cleave the Von Willebrand Factor (VWF) that circulates in the blood mediating platelets adhesion onto damaged blood vessels [1].

Ultra large VWF (UL-VWF) multimers are secreted as a reaction to a thrombogenic stimulus. ADAMTS13 converts VWF multimers into smaller fragments (dimers and monomers) with less activity. This cleavage step reduces the VWF coagulation activity, so clotting cascade will not proceed appropriately [2].

There was an elevation of VWF antigen level and activity, and VWF-collagen binding assay in corresponding to the reduced level of ADAMTS13 antigen in haematological malignancies as similar as other solid tumours. However, the changes in VWF assays were significantly more evident in patients with aggressive lymphomas than acute leukaemia patients. This may explain the occurrence of more venous thromboembolism in lymphoma patients than acute leukaemia patients [3].

There was a predictive role of ADAMTS13 activity for the prognosis in AML patients after bone marrow transplantation (BMT). Despite ADAMTS13 levels in AML were not as low as in thrombocytopenic purpura (TTP) patients, ADAMTS13 activity was lower than normal and played a role in disease progression due to increased VWF levels, so the risk of excessive platelet aggregation is higher [4].

IL-6 can be produced autonomously from AML blasts via autocrine mechanism, or from bone marrow niche including normal stromal cells or monocytes, or these latter cells can induce IL-6 production from blast cells via paracrine signalling [5–7]. High circulating IL-6 produced by AML blasts signify worse prognosis, reduced event-free survival and overall survival in adult and paediatric patients with AML [7–9]. IL-6 promotes relapse in AML paediatric patients via supporting the leukaemia stem cells (LSCs) through inducing a STAT3 downstream signalling, that might enhance the survival and expansion of LSCs in the initial rounds of chemotherapy; thus, disease relapse occurs [7, 10]. So, it was recommended to measure IL-6 for risk stratification in AML patients. Moreover, blocking IL-6 signalling pathways may eradicate chemo-resistant LSCs and prevent disease relapse [11].

Liu et al., assumed that inflammation may play a role in the reduced ADAMTS13 levels in AML patients. Also, lower level of ADAMTS13 was found in AML patients with infection owing to the inflammatory micro-environment [12].

A study by Lee et al. on 97 newly diagnosed intensively treated patients with acute myeloid leukaemia (AML) revealed that 65% of the patients did not have adequate circulating vitamin D levels. Furthermore, those patients had an increased association with worse prognosis [13].

Vitamin D has an immune-modulatory role as it inhibits the pro-inflammatory pathways (NK, Th1 and Th17 cells) and production of pro-inflammatory cytokines like IL-6, TNF-α, IL-2, and IFN-γ, Activin A, vascular endothelial growth factor (VEGF), osteopontin, calbindin, progesterone-induced blocking factor (PIBF) and induces anti-inflammatory pathways (Th2 and Treg cells) [14, 15].

Supplementation of vitamin D might help in preventing infections in the whole population, boosting their immune response, and modulating excessive inflammatory reactions through down regulation of IL-6 production [16].

In a recent review, Zhang and Liu suggested a possible association between vitamin D deficiency and vulnerability to infection with the highest rate for COVID-19 infection and deaths specifically in the Northern countries owing to the reduced vitamin D production via sunlight exposure [17, 18]. IL-6 was significantly increased in COVID-19 patients, so using an adequate source of vitamin D might help in reducing this pro-inflammatory response to infections [19].

Based on all the previous studies' findings, the relationship between (IL-6 and ADAMTS13), (IL-6 and vitamin D), the role of each of them in AML (pathogenesis and disease course), and the previous clinical trials for using vitamin D in treating AML; we investigated the effect of correcting serum vitamin D via vitamin D supplementation, and its effect on plasma IL-6 level and ADAMTS13 level along with the induction chemotherapy for AML patients.

## Material and methods

### Patients

The total studied subjects (52 adults > 18 years old) were divided into 3 groups; (Group A) 34 de novo AML patients including subgroups (A1):17 patients with (vitamin D deficiency < 20 ng/ml) without vitamin D therapy, (A2): 17 patients with vitamin D deficiency < 20 ng/ml and got extra vitamin D supplementary doses along with their ordinary induction chemotherapy cycle, and (Group B): 4 de novo AML patients without vitamin D deficiency / supplementation. Finally, (Group C): that included 14 healthy subjects (Table 1).

**Table 1:** Lab data of the case vs. control group units (1st day)

	Case group	Control group	*P*-value
No. (%)	38 (73%)	14 (27%)	N/A
Age (years)	41.13 ± 12.54	37.4 ± 13.5	0.371
Gender (M/F); No.(%)	21(55.3%)/ 17(44.7%)	8(57%)/6 (43%)	0.579
Sub groups	**†** Group A1; 17 (32.7%) **‡** Group A2; 17 (32.7%) **††** Group B; 4 (7.6%)	# Group C	
FAB classification	M1 = 3 (8%), M2 = 8 (21%), M3 = 5 (13%), M4 = 13 (34%), M5 = 8 (21%), M6 = 1 (3%)	N/A	
Erythrocyte sedimentation rate (ESR) (mm/1st hour)	69.5 (23–125)	8 (3–15)	0.0001*
Lactate dehydrogenase (LDH) (U/L)	612.5(169–3253)	224.5(130–332)	0.0001*
C-reactive protein (CRP) (mg/L)	88 (9–180)	7 (2–13)	0.0001*
Uric acid (mg/dL)	4.6 (2.4–11.5)	4.85 (1.8–7.1)	0.312
ALT (U/L)	20 (7–97)	22.5 (3–44)	0.244
AST (U/L)	24.5 (8–78)	24 (2–42)	0.807
Albumin (gm/L)	3.8 (2.70–4.4)	3.85 (3.4–4.7)	0.053
Creatinine (mg/dL)	0.8 (0.4–1.6)	0.6 (0.2–0.9)	0.028*
Total leucocytic count (TLC) (× 10^3^/ μL)	20.6 (2.4–229)	6.5 (4–10)	0.001*
Haemoglobin (gm/dL)	8.184 ± 1.65	13.33 ± 1.24	0.0001*
Platelet (× 10^3^/ μL)	27.5 (7–18)	284.5 (167–400)	0.0001*
Bone marrow blast %	66.45 ± 17.35	N/A	N/A
Prothrombin time (PT) (s)	14.88 ± 2.21	12.15 ± 0.97	0.0001*
Partial thromboplastin time PTT (s)	30.75 ± 6.11	31.14 ± 3.11	0.764

*Statistically significant

^†^Group A1: deficient vitamin D group (< 20 ng/ml) without vitamin D supplementation

^‡^Group A2: deficient vitamin D group (< 20 ng/ml) with vitamin D supplementation

^††^Group B: normal vitamin D (> 20 ng/ml)

^#^Group C: healthy subjects

M/F, male/ female; mm/1st hour, millimetre in the first hour; U/L, unit per litre; mg/L, milligramme per litre; mg/dL, milligramme per decilitre; gm/L, gramme per litre; μL, microliter

All subjects had no previous history of chronic diseases, autoimmune diseases, congenital thrombotic/ haemorrhagic diseases or malignancy and currently not pregnant or having thrombotic thrombocytopenic purpura. Study subjects were asked to provide written informed consent according to the Declaration of Helsinki of 1979.

The case group was diagnosed as AML after peripheral blood examination for blast cells, sternal bone marrow aspiration for direct bone marrow film examination, blast percent, and carrying out immune-phenotyping of the blast cells using flow cytometry (FACScan, Becton Dickinson, San Jose, California, USA) and conventional cytogenetic analysis.

All subjects were subjected to full history taking, clinical examination, routine laboratory investigations including CBC, AST, ALT, creatinine, uric acid, PT, PTT, INR, inflammatory markers (ESR, LDH,CRP), HCV Ab, HBsAg and HIV Ab.

On the 1st day of induction chemotherapy; 4.5 ml venous sample was taken and used as 2 ml blood on EDTA plasma (preserved in – 20 °C) for IL-6 and ADAMTS13 )Luminex Corporation, Luminex^®^ 200TM, Austin, USA), 2.5 ml on plain tube for serum vitamin D level (case group only) using (Roche Diagnostics, Cobas 6000 e 601, Switzerland).

Cholecalciferol 2800 I.U./ ml. was given as daily oral dose to the vitamin D-deficient subjects (17 cases) in (Group A2) for 28 days to correct the deficiency “according to the manufacturers recommendation for adults” concomitantly with the induction Chemotherapy cycle for AML treatment protocol. This therapeutic trial was approved by www.Clinicaltrials.gov with Identifier: NCT05149339.

On the 28th day (case group only), sternal bone marrow sample was aspirated for follow-up of the blast cells percent, 4.5 ml venous sample was taken and used as 2 ml blood on EDTA for IL-6 and ADAMTS13 (all cases) and 2.5 ml on plain tube for serum vitamin D level “therapy group only (A2)”.

### IL-6 and ADAMTS13 measurement

Multiplex Sandwich ELISA with Luminex^®^ Discovery Assay was used for IL-6 and ADAMTS13 measurement. “A human Premixed Multi-Analyte Kit for the simultaneous detection of multiple human biomarkers in plasma”; (USA R&D Systems; Bio-Techne Ltd.) using Luminex^®^ 200™.

**Fig. 1:**
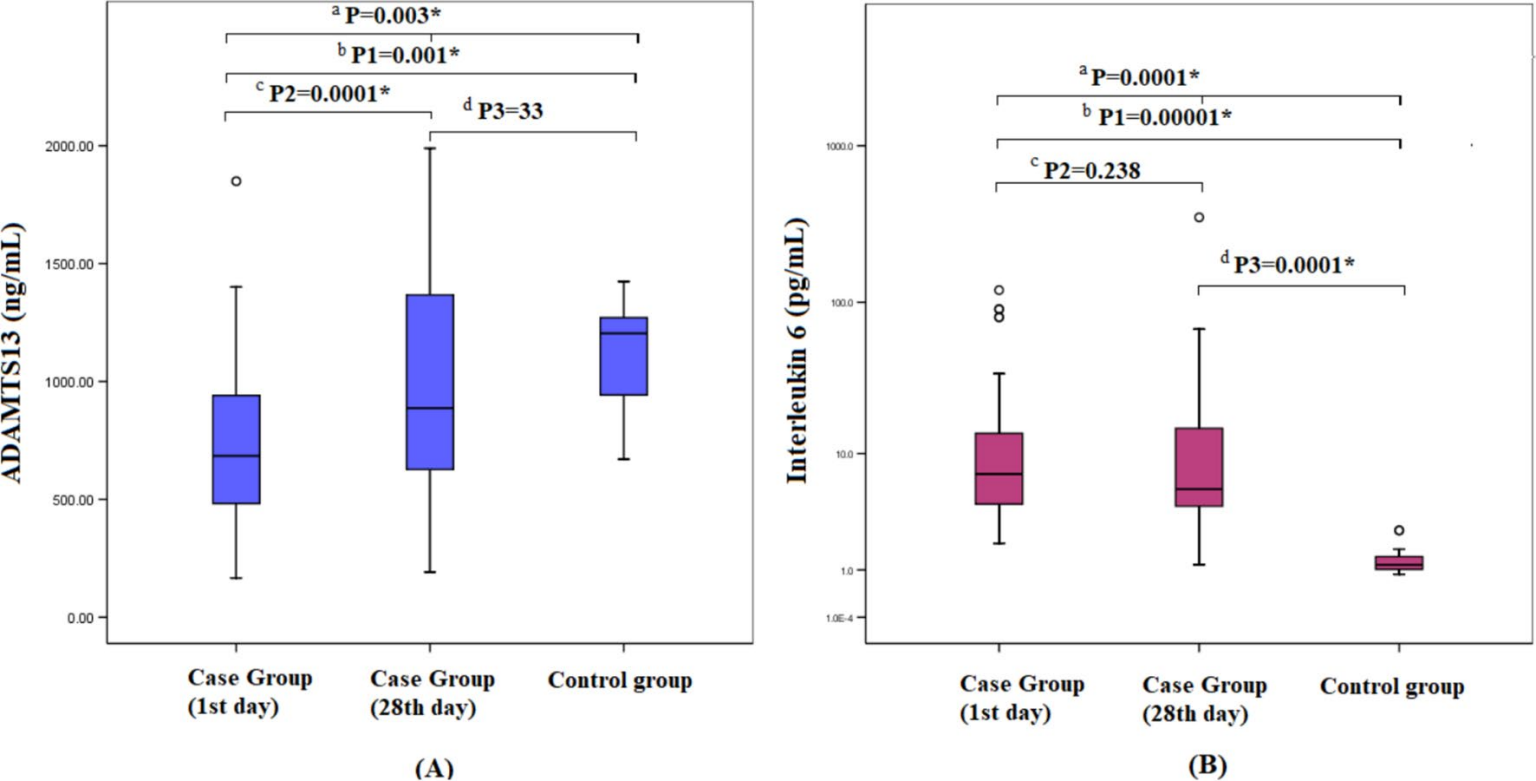
Boxplot presentation of ADAMTS13 and IL-6 among the case (1st and 28th) and control groups. **A** ADAMTS13; **B** IL-6

**Fig. 2:**
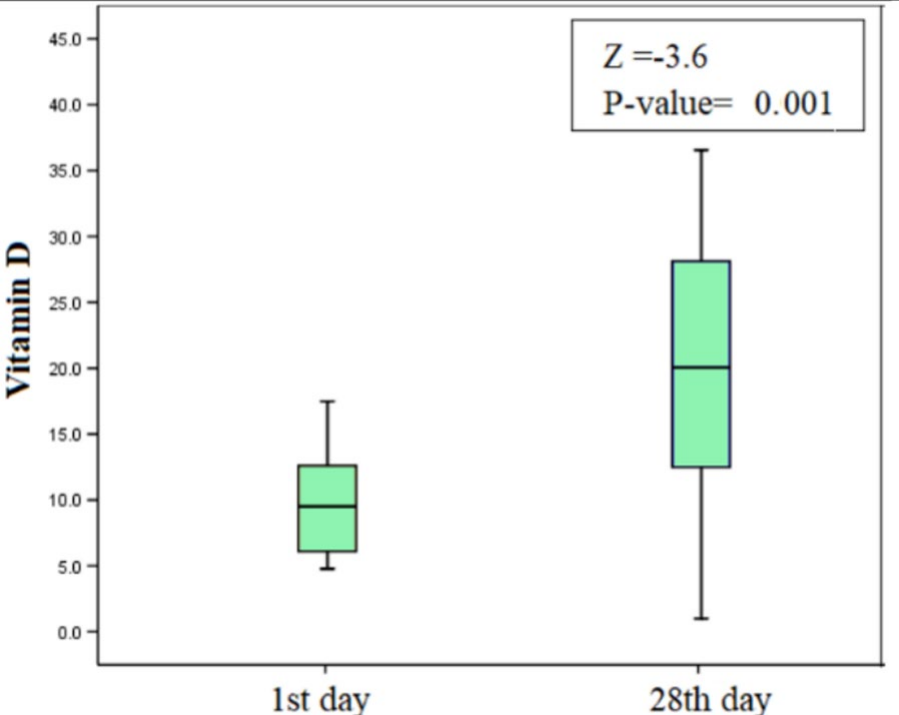
Boxplot presentation of vitamin D on both 1st day and 28th day of the induction chemotherapy among the group with vitamin D therapy

The assay was calibrated against highly purified recombinant human biomarkers produced at R&D Systems^®^. A standard curve for each analyte was created by computer software capable of generating a five parameter logistic (5-PL) curve-fit (Figs. 1, 2).

### Statistical analysis

All data were collected, tabulated using Microsoft Excel 365, and then statistically analysed using SPSS 25.0 for windows (SPSS Inc., Chicago, IL, USA). Quantitative variables were expressed as the mean ± SD & median (range).

Fisher's exact test was used for comparing categorical data. Non-parametric Mann–Whitney U (M–W) test was used to compare variables in case vs. control and between two case subgroups. While Kruskal–Wallis test (K-W) was used to compare a variable between more than two case subgroups. Spearman coefficient correlation was used to assess the correlation between ADAMTS13, IL-6, vitamin D and inflammatory markers. Wilcoxon test was used to compare the change of the previous biomarkers on the 1st and the 28th day of the induction chemotherapy. At confidence interval 95% and *P*-value of < 0.05; the applied test was considered statistically significant.

This was a case–control study held on 52 subjects including 38 patients diagnosed with de novo acute myeloid leukaemia (AML), and 14 healthy control subjects. A non-randomized controlled trial was applied onto the case group subjects. The study was carried out in the clinical pathology and medical oncology department of Zagazig University Hospitals from January 2019 to March 2020.

## Results

This study was a case–control study that was held in the Clinical pathology and Haemato-oncology departments in Zagazig University Hospitals from January 2018 to March 2020. Fifty-two adults aging more than 18 years were included. They were divided into two groups: 38 de novo AML cases and 14 apparently healthy controls.

Among the case group, a non-randomized controlled trial was held. It was subdivided into two groups: Group A with 34 patients with vitamin D deficiency (< 20 ng/ml) and Group B with 4 patients with normal vitamin D level. Group A further included 17 patients without receiving any vitamin D supplementation (A1) and 17 patients who were treated for their vitamin D deficiency along with the induction protocol for their AML by extra vitamin D supplementary doses (A2) (Table 1).

### Demographic data

The case group included 21 males and 17 females with age 41.13 ± 12.536 years, while the control group included 8 males and 6 females with age 37.4 ± 13.5 years. Cases have shown higher levels of ESR, LDH, CRP, creatinine levels compared to the control group with *p*-values of 0.0001, 0.0001, 0.0001 and 0.028, respectively. No significant difference was found between the two groups regarding uric acid, albumin, ALT and AST levels (Table 1).

Our AML case group was classified according to FAB classification; 3 patients with M0/M1, 8 patients with M2, 5 patients with M3, 13 patients with M4, 8 patients with M5 and only 1 case with M6 (Table 1).

There was lower haemoglobin level, platelet count and higher total leucocytic count and prothrombin time in the case vs. control group with *p*-values of 0.0001, 0.0001, 0.001and 0.0001, respectively. No significant difference was found between the two groups regarding partial thromboplastin time (Table 1).

### ***AML cases vs. control group***

The case group has shown significantly lower ADAMTS13 levels and higher IL-6 levels compared to the control group with *p*-values of 0.001 and 0.00001, respectively. There was a significantly higher levels of ADAMTS13 on the 28th day vs. 1st day samples with *p*-value of 0.0001. No significant difference was found in Serum IL-6 levels on both 1st and 28th days. A significant elevation of IL-6 level was found on the 28th day vs. the control group with median values 5.51 vs. 1.15 pg/mL and *p*-value of 0.0001. However, no significant difference was found regarding ADAMTS13 levels (Table 2; Fig. 1).

**Table 2:** ADAMTS13 and IL-6 for the case and control groups

Statistics	Case group		Control group
	(1st day)	(28th day)	
ADAMTS13 (ng/mL)			
Mean ± SD	736.32 ± 405.2	996.34 ± 481.66	1120 ± 239.5
Median (range)	684 (166.8–1849.5)	888(192–1989.5)	1205(670–1425)
Significance between groups*	^a^P = 0.003*, K–W = 11.78 ^b^P1 = 0.001*, M–W = − 3.302 ^c^P2 = 0.0001*, Z = − 4.634 ^d^P3 = 0.33, M–W = 0.97		
IL-6 (pg/mL)			
Mean ± SD	19.6 ± 30.03	19.89 ± 57.02	1.39 ± 0.55
Median (range)	7.19 (2–119.7)	5.51(1.2–349.3)	1.15 (0.9–2.6)
Significance between groups*	^a^P = 0.0001*, K–W = 31.7 ^b^P1 = 0.00001*, M–W = -5.29 ^c^P2 = 0.24, Z = − 1.18 ^d^P3 = 0.0001*, M–W = − 4.87		

*Statistically significant

K–W, Kruskal–Wallis, M–W, Mann–Whitney test, Z, Wilcoxon test

ng/mL, nano gramme per millilitre; pg/mL, pictogram per millilitre

^a^*P* case group (1st day) vs. case group (28th day) vs. control group using Kruskal–Wallis

^b^*P*1 case group (1st day) vs. control group using Mann–Whitney

^c^*P*2 case group (1st day) vs. case group (28th day) using Wilcoxon test

^d^*P*3 case group (28th day) vs. control group using Mann–Whitney

### Effect of vitamin D status on ADAMTS13 and interleukin 6 among case subgroups

There was a significantly higher levels of vitamin D after vitamin D supplementary doses on the 28th day vs. the 1st day with median 22.8 ng/ml vs. 9.5 ng/ml and *p*-value of 0.0001 (Table 3; Fig. 2).

**Table 3:** Vitamin D on 1st day vs. 28th day of the induction chemotherapy in deficient vitamin D group with vitamin D therapy

Deficient vitamin D group with treatment (< 20 ng/mL) N = 17		
1st day	Mean ± SD Median (range)	9.7 ± 4.3 9.5 (4.8–17.5)
28th day	Mean ± SD Median (range)	22.6 ± 10.4 22.8 (5.9–37.5)

*Z* = − 3.62

*P*-value = 0.001*

*Statistically significant**; **Z, Wilcoxon test

ng/mL, nano gramme per millilitre

On the 1st day of induction therapy, the case group included 34 patients with vitamin D deficiency (< 20 ng/ml) and 4 patients with normal vitamin D level. Vitamin D-deficient group has shown higher levels of IL-6 than normal vitamin D level group with *p*-value of 0.001. No significant difference was found between the two groups regarding ADAMTS13 level (Table 4).

**Table 4:** ADAMTS13 and IL-6 in vitamin D deficient group vs. vitamin D normal level group on 1st and 28th day of induction chemotherapy

	Deficient vitamin D (< 20 ng/mL)	Normal vitamin D (> 20 ng/mL)	*P*-value (M-W)
1st day			
No. (%)	34 (89.5%)	4 (10.5%)	
ADAMTS13 (ng/mL)	684 (166.8–1400.5)	1190 (249.4–1849.5)	0.74
IL-6 (pg/mL)	7.59 (2.5–119.7)	2.53 (2.0–5.7)	0.001*

	Deficient vitamin D (< 20 ng/mL)	Corrected vitamin D (> 20 ng/mL)	*P*-value (M-W)
28th day			
No. (%)	22 (59%)	16 (41%)	
ADAMTS13 (ng/mL)	1274 (192–1989.5)	709.33 (314–1483.9)	0.009*
IL-6 (pg/mL)	6.94 (1.4–349.3)	4.09 (1.2–41.1)	0.002*

*Statistically significant, M–W Mann–Whitney test

ng/mL, nano gramme per millilitre litre; pg/mL, pictogram per millilitre

On the 28th day, the case group included 22 patients with vitamin D deficiency (< 20 ng/ml) and 16 patients with corrected vitamin D level with therapy. Vitamin D deficient group has shown higher levels of ADAMTS13 and IL-6 than corrected vitamin D level group with *p*-values of 0.009 and 0.002, respectively (Table 4).

There was a significant elevation of ADAMTS13 on the 1st day vs. the 28th day in both vitamin D deficient groups either with/without vitamin D supplementary treatment with *p*-value of 0.017 and 0.001, respectively. In the group deficient in vitamin D with therapy, lower levels of IL-6 were reported on the 28th day compared to the 1st day with a *p*-value of 0.049 (Table 5).

**Table 5:** ADAMTS13, IL-6 on both 1st day and 28th day of the induction chemotherapy in deficient vitamin D group with and without treatment vs. normal vitamin D group

	Deficient vitamin D group (< 20 ng/mL)		Normal vitamin D group (> 20 ng/mL) *N* = 4
	Without vitamin D treatment *N* = 17	With vitamin D treatment *N* = 17	
ADAMTS13 (ng/mL)			
1st day	808(172–1401)	642 (167–1183)	784 (249–1850)
28th day	1274 (192–1786)	627(314–1648)	1138 (792–1990)
*P*-value	0.001*	0.017*	0.066
IL-6 (pg/mL)			
1st day	7.6 (3.8–119.7)	7.6 (2.5–80.2)	2.25 (2–2.5)
28th day	6.9 (1.4–349.3)	5.5 (1.2–67.1)	2.1 (1.4–4.1)
*P*-value (Z)	0 .776	0.049*	0.998

*statistically significant, Z Wilcoxon test

ng/mL, nano gramme per millilitre litre; pg/mL, pictogram per millilitre

### ADAMTS13, IL-6 and vitamin D in relation to patient remission

On Day 28, patients were considered to have achieved complete remission (CR) following normalization of their complete blood count, defined as hemoglobin levels > 11 g/dL for female patients and > 12 g/dL for male patients. Platelets ranging from 150 to 450 × 10^3^/μL and white blood cell count ranging from 4 to 11 × 10^3^/μL. In addition, bone marrow aspiration shows < 5% blast cells.

By the 28th day of the induction chemotherapy 14 patients (36.8%) have reached remission, 24 patients (63.2%) haven’t. No statistically significant difference was found between the two groups regarding ADAMTS13, IL-6 or vitamin D (Table 6).

**Table 6:** ADAMTS13, IL-6 and vitamin D in cases with remission vs. no remission on 28th day of the induction chemotherapy

	In remission (*n* = 14; 36.8%)	Not in remission (*n* = 24; 63.2%)	*P*-value
ADAMTS13 (28th day) (ng/mL)	713.6 (433.6–1648)	639.98 (314–1061.2)	0.58
IL-6 (28th day) (pg/mL)	5.5 (4.1–67.1)	4.25 (1.2–67.1)	0.44
Vitamin D (28th day) (ng/mL)	19.7 (5.9–37.5)	29.7 (5.9–34.7)	0.27

*Statistically significant, M–W, Mann–Whitney test

ng/mL, nano gramme per millilitre litre; pg/mL, pictogram per millilitre

### Correlations between ADAMTS13, IL-6, vitamin D vs. bone marrow blasts and inflammatory markers

On the 1st day, a statistically significant negative correlation was found between ADAMT13 and (CRP, ESR and LDH) on 1st day samples with *p*-values of 0.015, 0.011 and 0.038, respectively. Also, a statistically significant positive correlation was found between IL-6 and (CRP, ESR and LDH) with *p*-values of 0.004, 0.033 and 0.002, respectively. On 28th day, a statistically significant positive correlation was found between IL-6 and (CRP, ESR and LDH) with *p*-values of 0.032, 0.028 and 0.0001. Also, A statistically significant negative correlation was found between vitamin D and LDH with *p*-value of 0.024 (Table 7).

**Table 7:** Correlation between ADAMTS13/Il-6 and lab data (case group)

	1st day		28th day	
	*R*	*P*-value	*r*	*P*-value
ADAMTS13 vs. CRP	− 0.391	0.015*	− 0.123	0.463
ADAMTS13 vs. ESR	− 0.41	0.011*	− 0.233	0.56
ADAMTS13 vs. LDH	− 0.338	0.038*	0.118	0.480
ADAMTS13 vs. BM blast %	− 0.246	0.136	− 0.143	0.390
IL-6 vs. CRP	0.452	0.004*	0.349	0.032*
IL-6 vs. ESR	0.346	0.033*	0.367	0.028*
IL-6 vs. LDH	0.483	0.002*	0.603	0.0001*
IL-6 vs. BM blast %	− 0.043	0.795	0.246	0.136
Vitamin D vs. CRP	− 0.216	0.194	− 0.212	0.385
Vitamin D vs. LDH	− 0.204	0.219	− 0.514	0.024*
Vitamin D vs. ESR	− 0.088	0.599	− 0.388	0.053
Vitamin D vs. BM blast %	− 0.063	0.709	0.253	0.296
Vitamin D vs.IL-6	− 0.222	0.193	− 0.485	0.035*
Vitamin D vs. ADAMTS13	0.038	0.825	− 0.425	0.070
IL-6 vs. ADAMTS13	0.077	0.656	0.130	0.449

*statistically significant

*r*, Spearman correlation; CRP, C-reactive protein; LDH, lactate dehydrogenase; BM, bone marrow

## Discussion

Low ADAMTS13 levels is associated with acute leukaemia [12]. Elevated IL-6 levels were observed in many patients with pre-leukemic and leukemic conditions. AML blasts produce inflammatory cytokines including interleukin-6, that decreases colony-forming prospective of CD34 + cells and depletes the hematopoietic progenitors. High circulating interleukin-6 signify worse prognosis for patients with AML [4, 20, 21]. Interleukin-6, also, inhibits the ADAMTS13 actions, so UL VWFM accumulates increasing the risk of thrombosis [22].Vitamin D has an immune-modulatory role inhibiting the pro-inflammatory pathways and pro-inflammatory cytokines production like IL-6 [14, 15]. So, we to tried to assess whether supplementation of vitamin D might help modulating the inflammatory reactions occurring in AML patients.

Our AML patients have shown higher levels of IL-6 compared to the control subjects with median value 7.19 vs. 1.15 and *p*-value of 0.00001 (Table 2; Fig. 1). This was consistent with Ahmed et al., Liu et al. & Zhang et al. [12, 24, 25] This was explained by the secretion of numerous inflammatory markers, including IL-6, from AML blasts, which contribute to the depletion of normal hematopoietic progenitors and the induction of endothelial remodelling [21, 23, 26].

We also go along with Liu et al. and Zhang et al. regarding our finding that, AML patients had reduced level of ADAMTS13 compared to the healthy controls with median values 684 ng/mL vs. 1205 ng/mL and *p*-value of 0.001 (Table 2; Fig. 1). Our finding was revealed that ADAMTS13 level is relatively reduced during AML due to the increased inflammatory markers and infections associated with the disease course which increase the VWF multimers increasing the ADAMTS13 consumption or catabolism or inactivation by thrombin [12, 25].

There was no statistically significant difference between IL-6 results on the 1st vs. 28th days (Table 2; Fig. 1). Our results go along with Neggard et al. who found that, IL-6 levels in cancer patients “who didn’t reach complete remission” showed no significant difference before and after cancer therapy [27]. However, our results were inconsistent with Abd El-Hafez et al. who found a significant reduction of IL-6 levels after the induction chemotherapy for de novo AML patients [23]. The conflict may be explained by the possibility that not all patients had achieved complete remission by Day 28, and were still experiencing neutropenia associated with fever and, consequently, elevated IL-6 levels. Also, our results support this one more time as higher levels of IL-6 were reported on the 28th day compared to the control group with median values 5.5 pg/mL vs. 1.15 pg/mL and *p*-value of 0.0001.

On the other hand, we have found significantly higher levels of ADAMTS13 on the 28th day compared with 1st day samples with median values 887.8 ng/mL vs. 684 ng/mL and *p*-value of 0.0001 (Table 2; Fig. 1). In addition, no significant difference was found between ADAMTS13 level on 28th day and the control group (Table 2; Fig. 1) despite it was still lower on the 28th day. These results go along with Sun et al. & Zhang et al. findings [25, 28].

These findings were demonstrated by the secretion of angiogenic factors by the malignant blasts, those increase angiogenesis and endothelial activation, this consequently results in UL-VWF secretion into the plasma mostly of an endothelial origin. UL-VWF is now cleaved by ADAMTS13 leading to its consumption. However, after induction chemotherapy, the blast count decreases and so this axis is down-regulated; consequently, lower UL-VWF secretion, and by default the ADAMTS13 levels may reach the normal owing to patient remission with blast cell reduction to < 5% [29–32].

On the 1st day of the induction therapy, 34 AML patients (89.5%) were having vitamin D deficiency with serum level < 20 ng/ml. Only 17 of them have received vitamin D therapy to correct their deficiency. By the 28th day 16 patients (41%) have reached normal vitamin D serum (corrected with therapy).

There was a significantly higher levels of vitamin D on the 28th day than the 1st day in the group with vitamin D therapy with median values 22.8 vs. 9.5 and *p*-value of 0.001 (Table 3; Fig. 2), This proves the efficacy of the given vitamin D supplementation to correct the vitamin deficiency according to the manufacturer’s recommendation and the daily recommended doses. Also, this was consistent with the study carried by Anna et al. [33] in which a 2800 I.U/day oral dose was given to the intervention group, which resulted in a significant higher vitamin D level than the placebo group.

There was a significantly higher IL-6 level in de novo AML patients with vitamin D deficiency (< 20 ng/ml) than those with normal vitamin D level on the 1st day of the induction chemotherapy with median values 7.59 pg/ml vs. 2.53 pg/ml and *p*-value of 0.001(Table 4). Moreover, on the 28th day there was a significantly higher IL-6 level in the subjects with vitamin D deficiency (< 20 ng/ml) than subjects with normal vitamin D level with median values 6.94 pg/ml vs. 4.09 pg/ml and *p*-value of 0.002 (Table 4). Our findings go along with Manion et al. who proved higher IL-6 levels in HIV patients with vitamin D deficiency than patients with insufficient or normal vitamin D levels [34].

Significantly higher levels of IL-6 were found on the 1st day vs. 28th day in the group with vitamin D therapy with median values 7.6 vs. 5.5 and *p*-value of 0.049 (Table 5). But in the group without vitamin D correction, the difference was insignificant (Table 5). This profile goes also along with Manion et al. [34] these results demonstrate that vitamin D increases the level of VDR protein expression, decreases STAT1 and STAT3 activation, and consequently downregulates the production of pro-inflammatory cytokines, including IL-6 [35].

The previous results were also in alignment with many studies that proved the effect of vitamin D supplementation on lowering IL-6 levels; as lower vitamin D levels have resulted in higher IL-6 levels in obese and diabetic patients with subsequent reduction in IL-6 with vitamin D supplementation in the two studies [36, 37]. Also, Krishman et al. assumed that; vitamin D may contribute to anti-inflammatory actions with beneficial effects in several cancers [38]. In addition, another in vitro study by Zhang et al., showed that vitamin D doses have suppressed lipopolysaccharide-induced IL-6 and TNF-α production by human monocytes [39]. While, Salekzamani et al. have shown a reduction in E-selectin and VCAM-1 expression after 16 weeks of vitamin D supplementation and so, little intimal carotid vascular injury was observed in atherosclerotic patients. This was clarified by the observation that vitamin D reduces IL-6 levels, consequently lowering E-selectin and VCAM-1 levels [40].

On the 1st day of induction chemotherapy, there was higher levels of ADAMTS13 in the normal vitamin D group vs. the deficient vitamin D group with median values 1190 ng/mL vs. 684 ng/mL, however this difference was statistically insignificant (Table 4). This goes along with Cohen-Hagai et al. who found that there was no significant difference in ADAMTS13 activity between the normal vitamin D group and the deficient one in patients with diabetic nephropathy [41].

Contrasting to our expectations, on the 28th day there was significantly lower ADAMTS13 levels in patients with normal vitamin D level vs. patients with vitamin D deficiency with median values 709 ng/mL vs. 1274 ng/mL and *p*-value of 0.009 (Table 4).

Higher ADAMTS13 level on the 28th day vs. 1st day in the group with deficient vitamin D without therapy was found, with median values 1274 vs. 808 and *p*-value 0.001. However, lower level was found on the 28th day in the deficient group with vitamin D therapy with *p*-value 0.017. Also, no difference was found in the group with normal vitamin D from the start (Table 5).

So, all of our observation and its pattern discrepancy between the 1st and 28th days may conclude that; “there was no effect for vitamin D status on ADAMTS13 level as it differs independently from the vitamin D level”. Also, these give a clue to the insignificant correlation between ADAMTS13 level and vitamin D level either on the 1st or the 28th days of the induction chemotherapy.

We have observed a positive correlation between IL-6 and ESR on the 1st day with *r* = 0.346 and *p*-value = 0.033. Also, significant positive correlation was found between IL-6 and C-reactive protein (CRP) on the 1st and 28th day with *r* = − 0.452 and 0.349 and *p*-values of 0.004 and 0.032, respectively (Table 7). We were consistent with Möller et al. [42] This may be explained as the locally synthesized IL-6 in inflammatory conditions reaches the liver through the bloodstream, where it induces massive production of hepatic acute phase reactants such as C-reactive protein (CRP), serum amyloid A (SAA), fibrinogen, haptoglobin, and α1-chymotrypsin [43, 44].

On the other side there was a negative correlation between ADAMTS13 and CRP on the 1st day samples only with *r* = − 0.39 and *p*-value of 0.015 (Table 7) and this was consistent with Liu et al. [12] This finding may be explained by the elevated plasma VWF antigen/activity leading to a reduced plasma level of ADAMTS13 protein by consumption as a part of the pathophysiological inter-relationship between neoplasm and inflammation. In addition to lowered ADAMTS13 synthesis in the liver, it was shown that sepsis with its inflammatory markers results in an increased ADAMTS13 catabolism by various cleaving proteases like granulocyte elastase [45].

Also, a negative correlation was found between ADAMTS13 and LDH levels on the 1st day samples only with *r* = − 0.338 and *p*-value of 0.038 (Table 7). This was consistent with Henry et al. who proved this correlation in patients with acute kidney injury in SARS-Covid19 patients with the profile of low haemoglobin, elevated LDH and non-significant changes in D-dimer/prothrombin time; as they considered it as a thrombotic microangiopathy like phenomenon, where coagulopathy was infection-driven [13, 51, 52].

Also, Shaaban et al., carried out their study on AML patient to prove the poor prognostic role of high LDH levels at the time of diagnosis [46]. High LDH levels usually indicates tissue damage and high cellular turnover; this in turn releases large amounts of VWF antigen multimers to the site of injury that causes ADAMTS13 consumption and inhibition of its activity via thrombin [46–48].

There was a positive correlation between IL-6 serum and LDH on both 1st and 28th days samples. This finding goes along with Thomas et al. [49] However, no correlation was found between IL-6 and the bone marrow blast percentage either on the 1st day or 28th day samples (Table 7). This was against Thomas el a., who proved the presence of a positive correlation between IL-6 and blast count claiming that: “Myeloid blasts secrete IL-6 with presence of an autocrine blast response to IL-6 via IL-6 receptor expressed on AML blasts” [23, 49]. However, we were consistent with Stevens et al., who recently suggested the bone marrow niche as an extra source for IL-6 in AML paediatric patients, where IL-6 acts on the blasts via its receptor to play a role in protecting blasts from the chemotherapy-induced apoptosis [7].

No correlation was found between vitamin D and CRP either on the 1st or the 28th day, these results agree with Peterson et al. who found no correlation in the healthy women [50]. However, we are incongruent with Kruit et al. who found a significant negative correlation between vitamin D and CRP in both inflammatory and non-inflammatory diseases in a study carried out on 923 patients [51]. The discrepancy with our results may be explained by the low number of our studied subjects. Also, they investigated this correlation on a variety of diseases vs. only AML in our study.

There was a negative correlation between vitamin D level vs. LDH on the 28th day with *r* = − 0.514 and *p*-value of 0.024 (Table 7). We were consistent with Choi et al. [13] who proved that vitamin D supplementation modulates inflammatory responses from the muscle damage induced by high-intensity exercise in rats. We also go along with Pilch et al. who have found that LDH activity measured one hour after eccentric exercise was significantly higher in their control group with suboptimal vitamin D levels without vitamin D supplementation than subjects with suboptimal vitamin D levels and vitamin D supplementation; thus, confirming the role of vitamin D as a factor in controlling and modulating muscle cell damage caused by eccentric exercise [52].

No correlation was found between vitamin D and IL-6 on the 1st day samples (Table 7). This goes along with the findings of Sun et al. and Nagpal et al. who found no significant correlation in healthy adults [53, 54]. However, there was a significant negative correlation between IL-6 and vitamin D on the 28th day samples with *r* = − 0.485 and *p*-value of 0.035 (Table 7). Our findings agreed with Chen et al., who found a significant negative correlation in Chinese women attending to fertility clinics [55], and with Manion et al. who found this negative correlation in HIV patients [34].

The inconsistency in the results regarding the presence of a significant negative correlation between IL-6 and vitamin D on the 28th day but not the 1st day may be explained by the low number of the studied subjects. In addition, other factors may influence IL-6 levels in AML patients rather than STAT1/3 transcription downregulation by vitamin D [35], including the blast cell count and bone marrow niche micro-environment; that were incriminated in higher IL-6 levels in AML patients [7, 23, 49].

Unfortunately, we’ve found no correlation between IL-6 and ADAMTS13 either on the 1st or the 28th day (Table 7). We went along with Hyun et al. who found no significant correlation between them in patients with DIC [47]. But we were inconsistent with other studies that proved a negative significant correlation in AML patients with ADAMTS13 levels < 336 ng/ml only on the 1st day samples; [12] and in O blood group young adults [42]. This conflict in findings may be attributed to the small sample size and the possibility that patients had not fully recovered from neutropenic fever by Day 28, resulting in persistently elevated IL-6 levels despite normalized ADAMTS13 levels.

## Conclusion

De novo AML patients have shown elevated IL-6 levels and reduced ADAMTS13 levels. AML FAB-subtype has no impact on ADAMTS13 or IL-6 levels either on the 1st or the 28th day. By the 28th day of the induction chemotherapy, AML patients had normalized ADAMTS13 level. However, IL-6 showed no significant change between the 1st and the 28th samples. Vitamin D has an anti-inflammatory effect decreasing IL-6 in AML patients. Vitamin D status has no effect on ADAMTS13 level either on the 1st or the 28th day of the induction chemotherapy. We recommend applying this study on larger sample size. Patients may be followed up for longer periods to monitor remission. Further studies should be done to assess the effect of vitamin D on ADAMTS13 and inflammatory markers in patients with ALL.

## Data Availability

No datasets were generated or analysed during the current study.
